# Microenvironmental Ecology of the Chlorophyll *b*-Containing Symbiotic Cyanobacterium *Prochloron* in the Didemnid Ascidian *Lissoclinum patella*

**DOI:** 10.3389/fmicb.2012.00402

**Published:** 2012-11-30

**Authors:** Michael Kühl, Lars Behrendt, Erik Trampe, Klaus Qvortrup, Ulrich Schreiber, Sergey M. Borisov, Ingo Klimant, Anthony W. D. Larkum

**Affiliations:** ^1^Marine Biological Section, Department of Biology, University of CopenhagenHelsingør, Denmark; ^2^Plant Functional Biology and Climate Change Cluster, University of Technology SydneySydney, NSW, Australia; ^3^Singapore Centre on Environmental Life Sciences Engineering, School of Biological Sciences, Nanyang Technological UniversitySingapore; ^4^Department of Biomedical Sciences, Core Facility for Integrated Microscopy, University of CopenhagenCopenhagen, Denmark; ^5^Julius-von-Sachs Institut für Biowissenschaften, Universität WürzburgWürzburg, Germany; ^6^Department of Analytical and Food Chemistry, Technical University of GrazGraz, Austria

**Keywords:** *Prochloron*, symbiosis, microenvironment, photobiology, microsensor, bioimaging, didemnid ascidian, *Lissoclinum patella*

## Abstract

The discovery of the cyanobacterium *Prochloron* was the first finding of a bacterial oxyphototroph with chlorophyll (Chl) *b*, in addition to Chl *a*. It was first described as *Prochloron didemni* but a number of clades have since been described. *Prochloron* is a conspicuously large (7–25 μm) unicellular cyanobacterium living in a symbiotic relationship, primarily with (sub-) tropical didemnid ascidians; it has resisted numerous cultivation attempts and appears truly obligatory symbiotic. Recently, a *Prochloron* draft genome was published, revealing no lack of metabolic genes that could explain the apparent inability to reproduce and sustain photosynthesis in a free-living stage. Possibly, the unsuccessful cultivation is partly due to a lack of knowledge about the microenvironmental conditions and ecophysiology of *Prochloron* in its natural habitat. We used microsensors, variable chlorophyll fluorescence imaging and imaging of O_2_ and pH to obtain a detailed insight to the microenvironmental ecology and photobiology of *Prochloron in hospite* in the didemnid ascidian *Lissoclinum patella*. The microenvironment within ascidians is characterized by steep gradients of light and chemical parameters that change rapidly with varying irradiances. The interior zone of the ascidians harboring *Prochloron* thus became anoxic and acidic within a few minutes of darkness, while the same zone exhibited O_2_ super-saturation and strongly alkaline pH after a few minutes of illumination. Photosynthesis showed lack of photoinhibition even at high irradiances equivalent to full sunlight, and photosynthesis recovered rapidly after periods of anoxia. We discuss these new insights on the ecological niche of *Prochloron* and possible interactions with its host and other microbes in light of its recently published genome and a recent study of the overall microbial diversity and metagenome of *L. patella*.

## Introduction

The phylum Cyanobacteria harbors a diversity of morphotypes ranging from minute <1 μm unicellular forms (*Prochlorococcus* spp.) up to 100 μm thick filaments (*Oscillatoria* spp.) and large colonial cell aggregations visible by the naked eye (Palinska, 2008). Cyanobacteria are the only oxyphototrophs among prokaryotes and are considered inventors of oxygenic photosynthesis >2.5 billion years ago, major drivers in the formation of the first biological communities in the fossil record, i.e., microbial mats and stromatolites, as well as key architects of the present biosphere through their photosynthetic O_2_ production leading to an oxic atmosphere over geologic time scales. Cyanobacteria are also the only oxyphototrophs capable of N_2_ fixation, either in specialized cells (heterocysts) or via spatio-temporal modulation of their metabolic activity (Stal and Zehr, 2008), a trait that is employed by several protists, plants, and animals harboring symbiotic cyanobacteria (Adams, 2000; Lesser et al., 2004). In recent years, the analysis of cyanobacterial genomes has revealed a large degree of genetic exchange and plasticity inside this phylum (Zhaxybayeva et al., 2006), and cyanobacteria are regarded as prime candidates involved in the endosymbiosis leading to the evolution of algae and higher plants (Price et al., 2012).

Cyanobacteria also harbor species exhibiting unique characteristics very different from “typical” cyanobacteria. Most notably, this includes the prochlorophytes and the Chl *d*-containing cyanobacteria in the genus *Acaryochloris* that exhibit fundamental differences in terms of pigmentation, structure, and properties of their photosynthetic apparatus (Partensky and Garczarek, 2003; Larkum and Kühl, 2005; Kühl et al., 2007a). More recently, findings of the new chlorophyll *f* in cyanobacterial enrichments from stromatolites (Chen et al., 2010), and a widespread but yet uncultivated diazotrophic cyanobacterium without a functional PSII (Bothe et al., 2010) add on to the concept of cyanobacteria being a genetic melting pot and origin for a wide variety of photosynthetic adaptations (Larsson et al., 2011; Schliep et al., 2012). The ecology of such exotic cyanobacteria is not well understood, and in this study we focus on the ecology and habitat characteristics of the conspicuous symbiotic cyanobacterium *Prochloron* spp., which can be found and harvested in large quantities from their tunicate hosts (so-called didemnid ascidians) on coral reefs and mangrove systems but has resisted all cultivation attempts since its discovery in 1975 (Lewin and Cheng, 1989).

The taxonomy of *Prochloron* remains ambiguous. The type species was initially called *Synechocystis didemni* and later renamed *Prochloron didemni* (Lewin, 1977). Detailed comparative electron microscopy of different symbiont-host associations indicated several characteristic morphotypes hypothesized to represent different phylotypes of *Prochloron* (Cox, 1986). However, molecular data comparing sequences of the *Prochloron* *16S rRNA* gene obtained from different didemnid ascidians and geographic location indicate a fairly strong global similarity (Münchhoff et al., 2007) and a genomic study of *Prochloron* samples along a >5000 km transect in the Pacific showed >97% identity and strong synteny of *Prochloron* genomes (Donia et al., 2011a,b).

**Figure 1:**
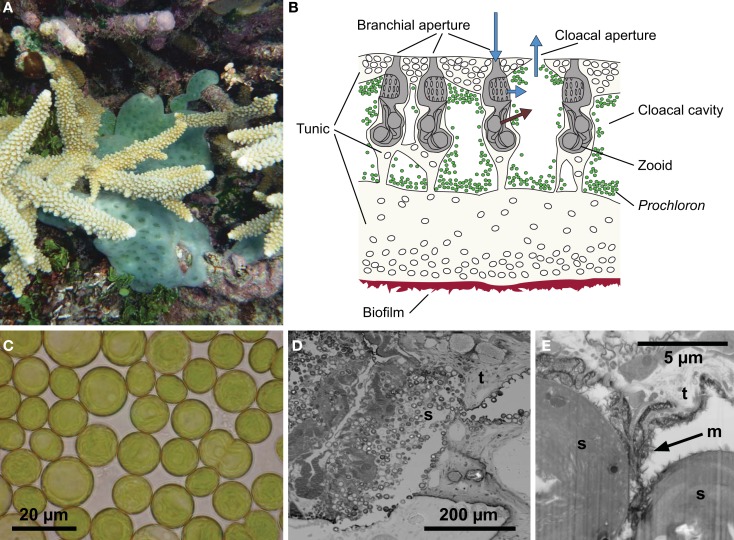
**The *Prochloron*-*Lissoclinum patella* symbiotic association**. **(A)** A colony (∼20 cm^2^) of the didemnid ascidian *Lissoclinum patella* covering corals with its 5–10 mm thick opaque cartilaginous tunic, i.e., a matrix of protein and cellulose-like carbohydrates containing calcareous spicules. **(B)** A schematic drawing of a cross-section illustrating the organization of zoids and symbionts in the tunic. The zooids are embedded in the tunic, where they suck in and filtrate particles out of the seawater. Waste products and filtered water are excreted into the surrounding peribranchial space and cloacal cavities in the colony tunic and eventually expelled via a joint cloacal aperture. **(C)** Individual *Prochloron* cells extracted from the ascidians, note the peripheral arrangement of thylakoids in the cells. **(D,E)** Electron micrographs **(D)** [TEM, **(E)** FIB SEM] of the symbiont-host interface in *L. patella* showing round *Prochloron* cells (s) embedded into the host tunic (t) and a fibrous exopolymeric substance (m, arrow). **(C)** Was redrawn and modified after Maruyama et al. (2003).

Cyanobacteria in the genus *Prochloron* are ∼7–25 μm wide (Cox, 1986), bright green spherical cells with stacked thylakoids oppressed to the cell periphery (Figure 1); they were discovered in close association with (sub)tropical didemnid ascidians (Lewin and Cheng, 1975; Newcomb and Pugh, 1975). While the presence of microbial phototrophs and O_2_ production in didemnid ascidians was well known (Maurice, 1888; Smith, 1935; Tokioka, 1942), it was a surprise that *Prochloron* contained no phycobilins but Chl *b* in addition to Chl *a* (Lewin and Withers, 1975). At that time, Chl *b* was only known from algae and higher plants, and *Prochloron* was for some time regarded a missing link in chloroplast evolution. However, molecular phylogenetic studies have unequivocally shown that *Prochloron* is but one of several lineages in the cyanobacteria, wherein Chl *b* has evolved (Palenik and Haselkorn, 1992; La Roche et al., 1996).

Along with the other two groups of prochlorophytes, i.e., *Prochlorococcus* and *Prochlorothrix*, *Prochloron* shares several additional differences to other cyanobacteria, including special membrane-bound Chl *a*/*b* light harvesting complexes that are unrelated to the Chl *a*/*b* antenna in the light harvesting complex of eukaryotic oxyphototrophs (La Roche et al., 1996; Partensky and Garczarek, 2003). Interestingly, genes encoding for the so-called “prochlorophyte Chl *b*-binding” proteins (*Pcbs*) are found both in prochlorophytes and in the Chl *d*-containing cyanobacterium *Acaryochloris marina*, which is not closely related to the prochlorophytes on basis of their *16S* *rRNA* gene phylogeny, indicating a more ancient origin of these genes or lateral gene transfer (Chen et al., 2005).

*Prochloron* forms symbiotic associations with didemnid ascidians in the genera *Didemnum*, *Trididemnum*, *Lissoclinum*, and *Diplosoma* and represents the only known obligate photosymbiosis in the phylum Chordata (Hirose et al., 2009a). Studies of the *18S rRNA*-based molecular phylogeny of didemnid ascidians indicate that photosymbiosis with *Prochloron* has occurred independently in each of these genera (Yokobori et al., 2006). Besides the unsuccessful attempts to cultivate *Prochloron* separated from its hosts, a symbiotic relation is supported, e.g., by observations of carbon and nitrogen exchange between *Prochloron* and host (Lewin and Pardy, 1981; Kremer et al., 1982; Griffiths and Thinh, 1983; Koike et al., 1993), light-enhanced growth of didemnids with *Prochloron* (Olson, 1986), and fascinating specializations in didemnids ensuring vertical transmission of *Prochloron* to ascidian larvae during maturation and before they leave the parent tunic (Hirose, 2009), i.e., the extracellular matrix of protein and cellulose-like carbohydrates, wherein the host zoids are embedded. However, the exact nature of the symbiosis and mutual benefits in the ascidian-*Prochloron* association is still poorly understood (Hirose and Maruyama, 2004).

Different modes of association between *Prochloron* and didemnid ascidians have been described (Cox, 1986; Hirose et al., 2009a) including (i) colonization of the outer surface and upper tunic, (ii) colonization of the inner cloacal cavities and the peribranchial space of zoids, (iii) a more wide spread colonization in the tunic, and (iv) intracellular *Prochloron* in some didemnid species. Additionally, there is macro- and microscopic evidence that *Prochloron* can be associated with the surface of holothurians (Cheng and Lewin, 1984), sponges (Parry, 1986), and a range of non-didemnid ascidians (Cox, 1986). Significant amounts of suspended *Prochloron* in seawater have been observed (Cox, 1986) and molecular surveys indicated that *Prochloron* could be thriving in stromatolites (Burns et al., 2004), but confirmation of such potentially free-living *Prochloron* awaits further direct evidence of their actual presence and ecological niche in such systems.

Detailed physiological and photobiological studies of *Prochloron* are few (reviewed in Kühl and Larkum, 2002) and have mostly involved measurements on solute exchange or photosynthetic performance of intact ascidian-*Prochloron* associations or on extracted *Prochloron* cells, which only remain photosynthetically competent for a few hours to a day (Critchley and Andrews, 1984). This has hampered both cultivation attempts and a more detailed understanding of the ecological niche of *Prochloron* *in hospite*. However, some niche characteristics are well known such as the presence of UV absorbing mycosporin-like compounds that act as sunscreens (Dionisio-Sese et al., 1997; Maruyama et al., 2003; Hirose et al., 2006), the presence of enzymes for detoxification of reactive oxygen species (ROS; Lesser and Stochaij, 1990), and the prevalence of a diversity of highly bioactive secondary metabolites (especially cyanobactins) in *Prochloron* that may, e.g., have allelopathic functions against other phototrophs (Schmidt and Donia, 2010; Donia et al., 2011b; Schmidt et al., 2012).

The photosynthetic apparatus of *Prochloron* appears similar to other cyanobacteria with conventional electron transport reactions through photosystem (PS) I and II and a typical oxygen evolution complex. However, special light harvesting supercomplexes based on the prochlorophyte chlorophyll *a/b* (pcb) protein are present in both PSI and PSII (Bibby et al., 2003; Murray et al., 2006). These light harvesting systems give rise to a highly efficient photosynthetic electron transport system, where the maximum quantum yield of PSII approaches 0.82, similar to that in higher plants and much higher than in most cyanobacteria (Schreiber et al., 1997, 2002).

Carboxysomes are present, and a significant part of the carbonic anhydrase present is found in these bodies (Griffiths, 2006) along with all the Rubisco enzyme (Swift and Leser, 1989), which is responsible for fixing CO_2_ to phosphoglyceric acid. There appears to be an active supply of organic products to the ascidian host and these appear to be early carbon products of the Calvin–Benson cycle (Kremer et al., 1982). This translocation is host-dependent and represents about 12–56% of reduced carbon for host respiration (Olson and Porter, 1985; Alberte et al., 1987).

Other aspects of metabolism, e.g., the source and dynamics of N_2_ fixation and nitrogen turnover in the ascidian-*Prochloron* symbiosis are still unresolved and debated (e.g., Odintsov, 1991; Donia et al., 2011b). There is evidence that in addition to carbon, nitrogen is also recycled in the symbiosis (Koike et al., 1993). Ammonium is the major nitrogenous waste of the host (Goodbody, 1974) and is taken up by *Prochloron* (Parry, 1985). It has been proposed that N_2_ fixation contributes to the nitrogen requirements (Paerl, 1984). This was disputed by Parry (1985), but is supported by nitrogen isotope ratios in host and *Prochloron*, which are consistent with nitrogenase activity (Kline and Lewin, 1999).

The recent publications of the first draft *Prochloron* genome as well as studies of the microbiome of photosymbiotic didemnids (Donia et al., 2011a,b; Behrendt et al., 2012a) have now added significant new insights to the ecology of *Prochloron*, and potential interactions with its didemnid hosts and other members of the microbiome. However, the interpretation of such molecular surveys in terms of physiology and metabolic interactions mostly rely on genomic evidence that still needs experimental verification for *Prochloron* under natural conditions.

While many studies have speculated about the microenvironmental conditions of *Prochloron* *in hospite*, very few experimental data on the microenvironment in didemnid ascidians have been published (Kühl and Larkum, 2002; Behrendt et al., 2012a). These data indicate highly dynamic physico-chemical conditions that are strongly modulated by irradiance. In this study, we present new data and review the current knowledge about the microenvironment and metabolic activity of symbiotic *Prochloron* associated with didemnid ascidians. We focus on the large colonial didemnid ascidian *Lissoclinum patella*, which covers significant areas of substratum on coral reefs with an opaque cm-thick cartilaginous tunic harboring large quantities of *Prochloron* in the internal peribranchial and cloacal cavities of the filter-feeding zoids (Figure 1). The tunic of *L. patella* is an integumentary extracellular matrix of protein and cellulose-like carbohydrates covered by a dense tunic surface cuticle containing calcareous spicules as well as a diversity of specialized tunic host cells (Goodbody, 1974; Hirose, 2009).

Using microsensors and advanced bioimaging, we present detailed data on the physical and chemical boundary conditions *in hospite* and show how the metabolic activity of *Prochloron* and its host modulate the microenvironmental conditions in response to changes in irradiance. We discuss these new insights into the ecological niche of *Prochloron* and possible interactions with its host and other microbes in light of its recently published genome and recent studies of the overall microbial diversity and metagenome of the didemnid ascidian host *L. patella*. (Donia et al., 2011a,b; Behrendt et al., 2012a).

## Results

### Distribution and photosynthetic activity of *Prochloron*

**Figure 2:**
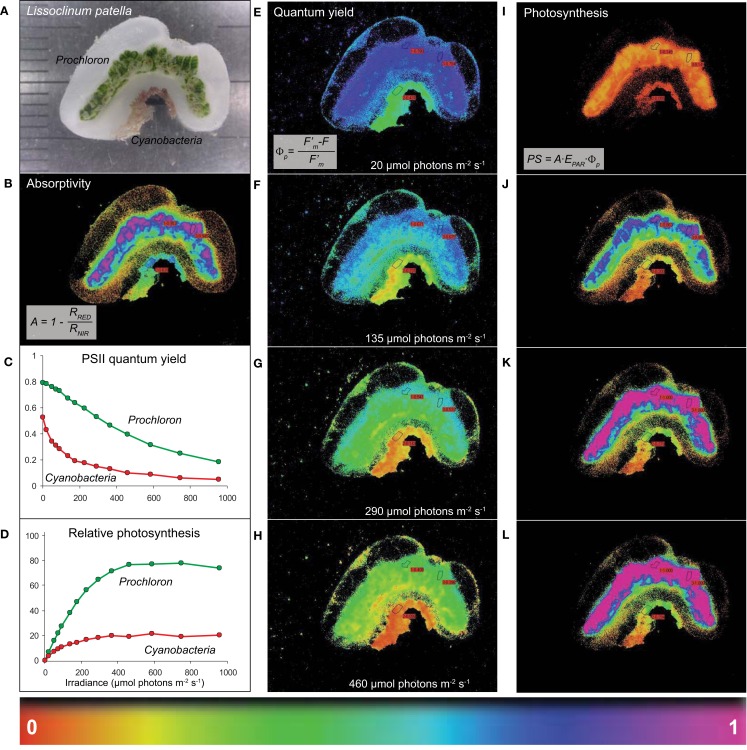
**Imaging of light absorption and relative photosynthetic activity of *Prochloronin hospite***. **(A)** A cross-section of *L. patella* showing a bright green layer of *Prochloron* cells in the cloacal cavities and the peribranchial space of the zooids and a reddish cyanobacterial biofilm colonizing the underside of the basal tunic. Light scattering spicules are embedded in the tunic, especially in the upper tunic and immediately below the *Prochloron* layer. **(B)** Absorptivity of red light (λ_max_ 650 nm) in the different layers of *L. patella*. **(C,D)** Quantum yield of PSII and derived relative photosynthesis vs. irradiance curves for regions of interest (ROI) in the *Prochloron* layer and the cyanobacterial biofilm colonizing the underside of the ascidian. **(E–H)** Spatial distribution of PS II quantum yield and the derived relative photosynthesis **(I–L)** in *L. patella* at selected incident irradiance levels, *E*_PAR_. A proxy for the relative photosynthetic activity was calculated from the relative PSII electron transport rate rETR = Φ_PSII_ *E*_PAR_, taking the different absorptivity over the imaged specimen into account, as PS = *A* Φ_PSII_ *E*_PAR_.

Both macroscopic imaging and electron microscopy of *L. patella* sections showed a dense colonization of the cloacal cavities and the peribranchial space of zooids by apparently intact and dividing *Prochloron* cells (Figures 1 and 2A). *Prochloron* was closely associated to the host tunic, either directly embedded in the tunic or anchored in an exopolymeric substance apparent in the electron micrographs (Figures 1D,E).

High absorptivity of red light was observed in the densely pigmented *Prochloron* layer and the cyanobacterial biofilm underneath the ascidian, while more faint light absorption was observed associated with the tunic matrix especially at the colony surface and immediately below the *Prochloron* layer (Figure 2B). *Prochloron* exhibited a high maximum PSII quantum yield of ∼0.8 after dark acclimation *in hospite*, while the effective PSII quantum yield declined down to ∼0.2 with increasing levels of incident irradiance (Figure 2C). The cyanobacterial biofilm exhibited a lower maximum PSII quantum yield of ∼0.5–0.6 decreasing to an effective PSII quantum yield of <0.1 at the highest irradiance. Using the product of absorptivity, quantum yield and incident irradiance as a proxy for photosynthetic activity, the *Prochloron* and cyanobacterial biofilm also showed different acclimation to irradiance (Figure 2D), while more faint activity was found in a thin layer on the tunic surface and a layer immediately below the *Prochloron* cells. The cyanobacterial biofilm exhibited onset of saturation, as determined from the intersection of the initial slope and the maximal activity level, at an irradiance of ∼100 μmol photons m^−2^ s^−1^, while the *Prochloron* layer showed saturation of photosynthesis at irradiances >250 μmol photons m^−2^ s^−1^. Both zones exhibited no photoinhibition due to short exposure to irradiances of ∼1000 μmol photons m^−2^ s^−1^. Imaging of the effective quantum yield of PSII and the derived relative photosynthesis revealed further details on the distribution of photosynthetic activity in *L. patella* (Figures 2E–L). The activity distribution in the cloacal and peribranchial parts of *L. patella* closely followed the distribution of the green colored areas indicative of *Prochloron* and the pink-colored cyanobacterial biofilm (compare, e.g., Figures 2A,I).

### Light penetration and attenuation in *L. patella*

**Figure 3:**
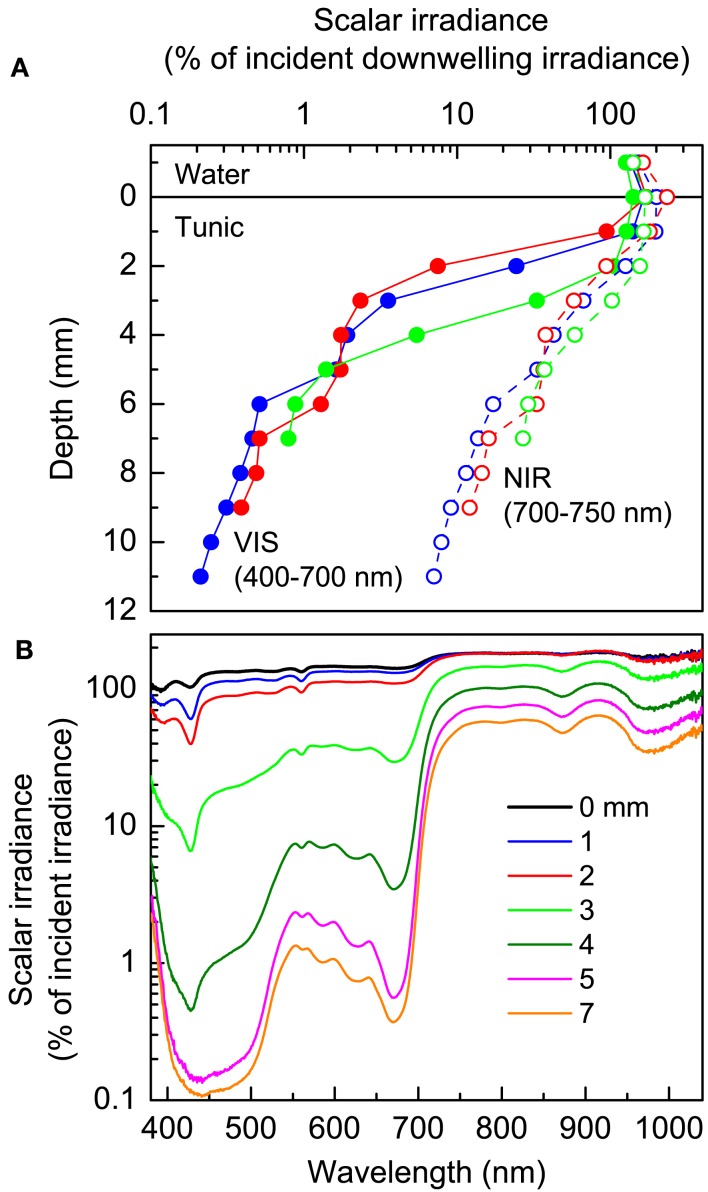
**Light propagation in *L. patella***. **(A)** Depth profiles of integrated scalar irradiance (in % of the incident downwelling irradiance at the tunic surface) for VIS (VIS, 400–700 nm) and the part of near-infrared radiation (NIR, 700–750 nm) which can potentially drive oxygenic photosynthesis via Chl *d* or *f*. **(B)** Spectral scalar irradiance (in % of the downwelling spectral irradiance at the tunic surface) measured at increasing depth below the tunic surface (given as numbers on curves).

Scalar irradiance measurements in intact *L. patella* specimen revealed both a pronounced scattering of incident light in the upper tunic as well as strong attenuation of visible light (VIS; 400–700 nm) in the deeper layers (Figure 3A). VIS was strongly scattered in the uppermost tunic leading to a local increase of scalar irradiance reaching ∼140–170% of incident light. About 10–40% of the incident VIS penetrated the upper tunic, while strong attenuation in the *Prochloron*-containing layer (ranging in depth from ∼1.5–2 to ∼4–6 mm below the tunic surface) reduced VIS down to only a few % of the incident irradiance (Figure 3A). In the lower part of the tunic, attenuation of VIS was less strong.

Near-infrared radiation (NIR) showed much less attenuation but strong scattering in *L. patella* (Figure 3A). The scalar irradiance of NIR (700–750 nm) reached ∼170–230% of downwelling NIR in the uppermost tunic. About 30–40% of the incident NIR still prevailed below the *Prochloron* layer and NIR was less strongly attenuated in the lower part of the tunic.

The scalar irradiance spectra showed further details on the propagation and attenuation of light in *L. patella* (Figure 3B). In the NIR region, weak minima around 800–805 and 870–880 nm indicated the presence of some BChl *a*, while a stronger minimum at 965–985 nm may indicate the presence of another BChl-like photopigment. However, NIR was much less attenuated than visible wavelengths and combined with strong scattering this led to significant local enhancement of the scalar irradiance in the uppermost tunic.

In the visible spectral region, scalar irradiance showed strong spectral minima corresponding to absorption maxima of several photopigments: a strong minimum at 675 nm and a shoulder at ∼650 nm revealed the presence of Chl *a* and Chl *b* indicative of *Prochloron*: a minimum at ∼625 nm, as well as minima at 585–590, ∼565, and a shoulder at ∼490–495 nm indicated presence of phycobiliproteins in *L. patella*. In the upper tunic, distinct minima at ∼390 and ∼426 nm could indicate the presence of photoprotective pigments, but we did not confirm the type or quantity of such compounds in this study.

### Distribution and dynamics of O_2_

**Figure 4:**
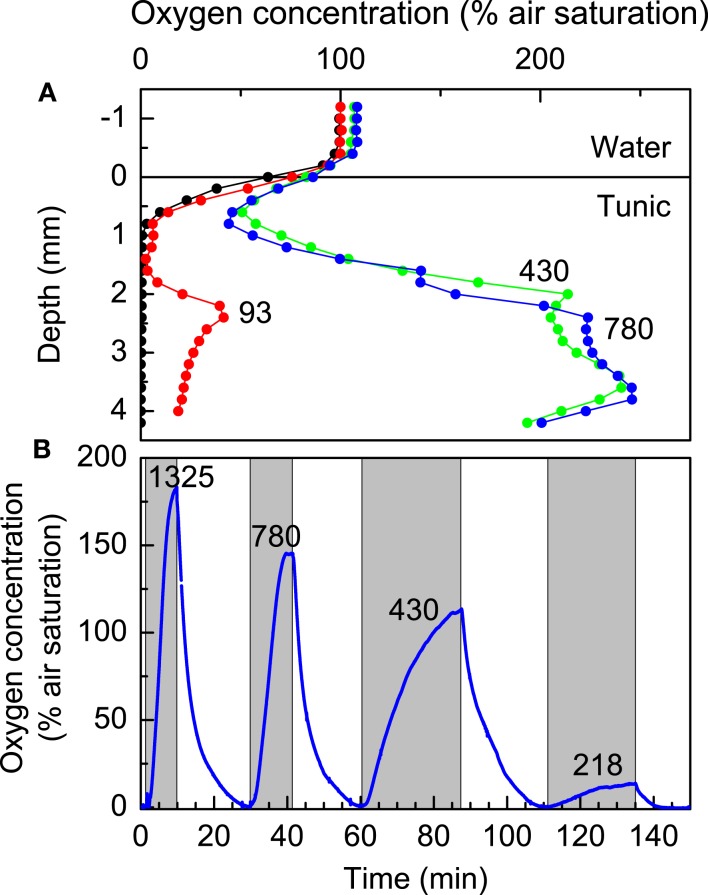
**Oxygen dynamics in *L. patella***. **(A)** Depth profiles of O_2_ concentration (in % air saturation) at increasing irradiance levels (given as numbers on curves in units of μmol photons m^−2^ s^−1^). **(B)** Dynamics of O_2_ concentration at ∼3 mm depth in a *L. patella* specimen under experimental light-dark shifts at different irradiance levels (given as numbers on curves in units of μmol photons m^−2^ s^−1^). Shaded areas indicate periods of illumination.

Profiling with thin O_2_ microelectrodes revealed a highly dynamic microenvironment of *Prochloron* inside *L. patella*, which was strongly regulated by ambient irradiance levels (Figure 4). In the dark, only the uppermost part of the test remained oxic, while the interior of *L. patella* was anoxic down to about 4 mm depth into the ascidian colony, although the surface was exposed to flowing aerated seawater in the flow chamber. Upon exposure to light, the ascidian showed rapid accumulation of O_2_ in its interior cavities due to intense photosynthesis in the densely populated *Prochloron* biofilm lining the peribranchial space and the internal cloacal cavities, while the upper tunic exhibited O_2_ consumption both in darkness and light (Figure 4A). Under an irradiance of 93 μmol photons m^−2^ s^−1^, O_2_ started to accumulate ∼2 mm below the tunic surface, where the microsensor entered the *Prochloron* layer; further, into this layer the declining O_2_ concentration indicated light limitation and net O_2_ consumption. At higher irradiance O_2_ increased rapidly and approached saturating levels reaching ∼250% air saturation in the *Prochloron* layer at irradiances >300 μmol photon m^−2^ s^−1^.

The O_2_ dynamics inside the ascidian was strongly affected by irradiance. Experimental light-dark shifts with the O_2_ microsensors fixed ∼3 mm below the ascidian surface in the *Prochloron* layer showed alternation from anoxic to hyperoxic conditions over time scales ranging from 15 to 30 min, where the rate of O_2_ build-up increased with irradiance indicative of an increased photosynthetic rate (Figure 4B). Even after prolonged time in darkness and anoxia, *Prochloron* photosynthesis exhibited an immediate onset of O_2_ production upon illumination. However, during profiling we often observed that the O_2_ level in the uppermost tunic of *L. patella* decreased over time upon repeated profiling, especially under high irradiance (data not shown), and such apparent wound reactions caused long term downward drift in many measurements and limited the amount of O_2_ profiles obtained.

**Figure 5:**
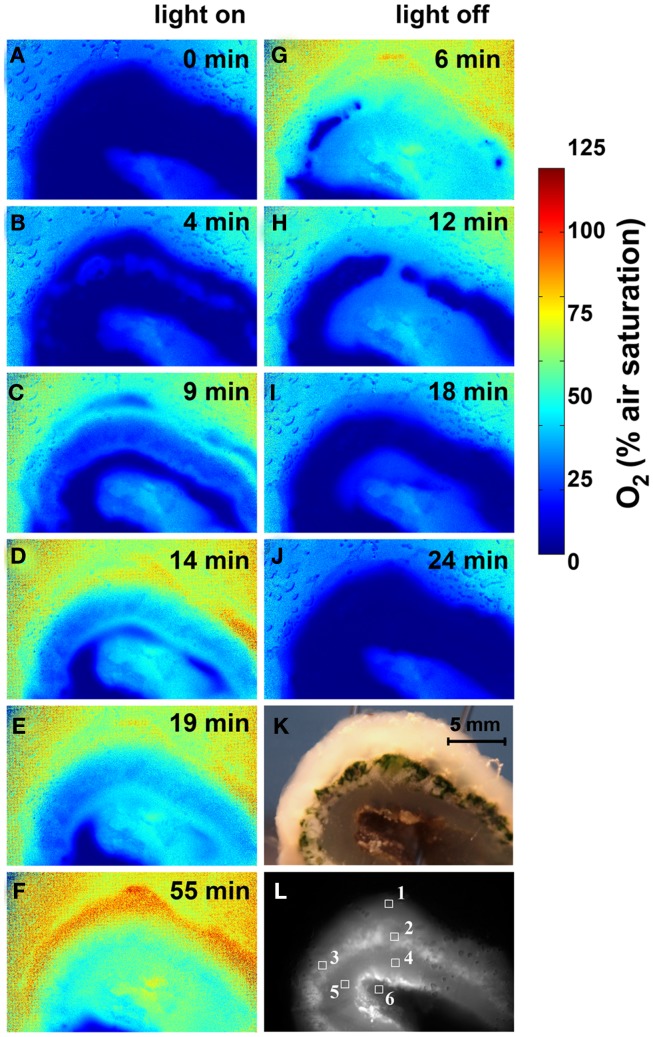
**Imaging of O_2_ concentration dynamics in *L. patella***. **(A–J)** Pseudocolor images of the O_2_ distribution in *L. patella* under an incident photon irradiance of 250 μmol photons m^−2^ s^−1^ and after darkening. Times in light and after darkening are indicated in minutes in each panel. **(K)** Photograph of *L. patella* cross-section. **(L)** Image of cross-section illuminated with red light showing the *Prochloron* layer and the biofilm underneath the ascidian. Regions of interest (ROI) show the origin of data plotted in Figure 6.

While the O_2_ microsensors only provided a limited number of spot measurements in the ascidians (Figure 4), the O_2_ dynamics were confirmed by imaging of O_2_ concentration across a *L. patella* specimen sliced vertically and pressed up against an O_2_ sensitive foil mounted inside the transparent flow chamber (see Materials and Methods for further details). This O_2_ imaging revealed similar dynamics in *L. patella* as observed in the microsensor data, showing anoxia in the *Prochloron* layer in darkness and enhanced O_2_ due to onset of photosynthesis upon illumination (Figure 5). However, the actinic light source used in the imaging setup only allowed homogenous illumination of the ascidian with a maximal photon irradiance of 250 μmol photons m^−2^ s^−1^ and this did not enable high super saturating O_2_ levels to be reached.

**Figure 6:**
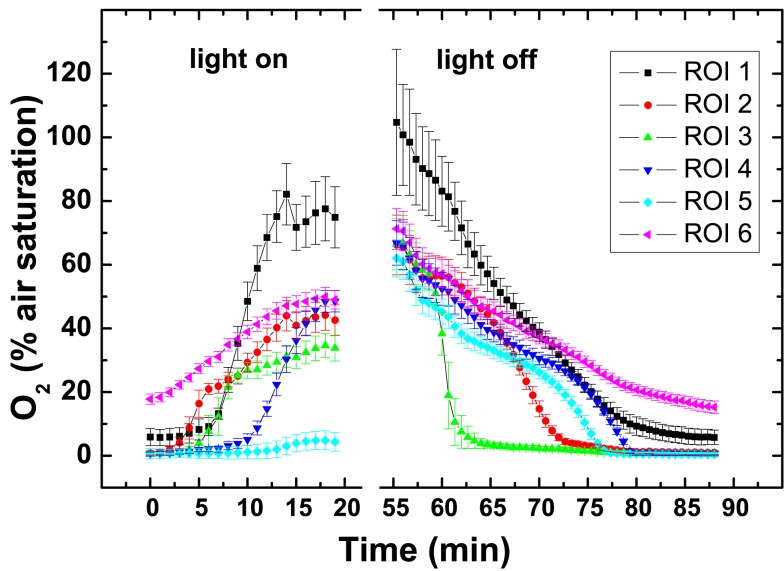
**Dynamics of O_2_ concentration in selected regions of interest (ROI; see Figure 5L) after on set of illumination with a photon irradiance of 250 μmol photons m^−2^ s^−1^ and after darkening**. Symbols represent the average ± standard deviation in each ROI.

Oxygen imaging revealed several zones in the ascidian contributing to the build-up of O_2_ (Figures 5C–E). Initially and shortly after onset of illumination, O_2_ evolution was detected in separate zones comprising the upper tunic surface, the *Prochloron* layer and the cyanobacterial biofilm layer on the ascidian underside (Figure 5D). As the O_2_ levels continued to increase and oxygenate the ascidian tissue, these distinct zonations between the test surface and the *Prochloron* layer became less distinct, while a second zone of O_2_ production just below the *Prochloron* layer and above the cyanobacterial biofilm became visible (Figures 5F,G). Upon darkening, the *Prochloron* layer exhibited the fastest O_2_ depletion, while the other regions in *L. patella* approached anoxia more gradually (Figures 5I–L). The upper tunic and the biofilm on the underside of *L. patella* remained oxic in darkness, albeit at low levels of ∼10–20% air saturation (Figure 5; ROI 1 and 6 in Figure 6).

### Distribution and dynamics of pH

pH is another chemical parameter in the ascidian host that potentially is strongly affected by *Prochloron* photosynthesis due to their CO_2_-fixation. Attempts to profile pH with glass needle microelectrodes were not successful due to their fragility and apparent local release of acid vacuoles in the ascidian tunic when inserting the relatively large needle electrodes (data not shown). Therefore, we adapted a new pH imaging approach to resolve the pH dynamics in *L. patella*. As this is the first time such imaging has been applied, detailed data on sensor performance, and calibration are given in the methods section at the end of this article.

**Figure 7:**
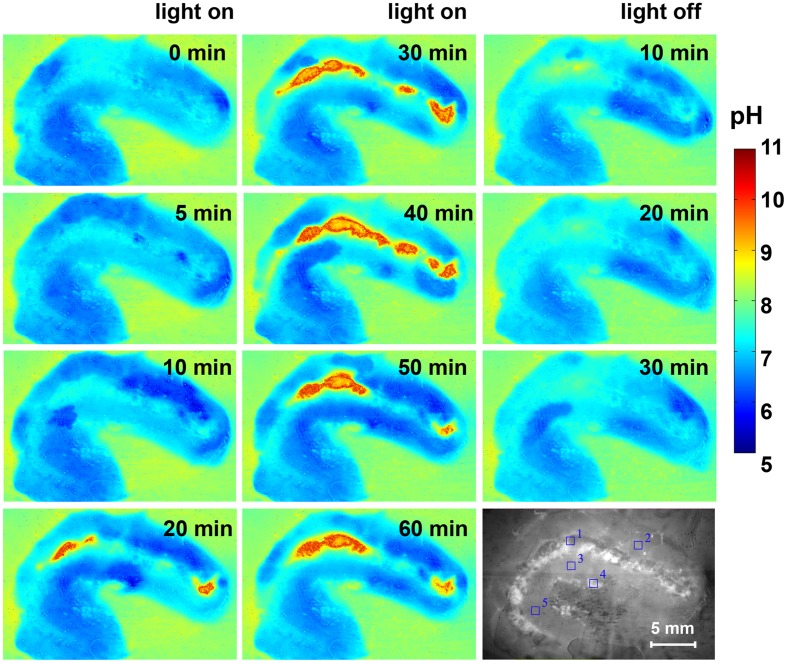
**Pseudocolor images of the pH distribution in *L. patella* under an incident photon irradiance of 250 μmol photons m^−2^ s^−1^ and after darkening**. Times in light and after darkening are indicated in minutes in each panel. The monochrome image (lowermost right panel) is the cross-section illuminated with red light showing the *Prochloron* layer and the biofilm underneath the ascidian. Regions of interest (ROI) are outline din blue and show the origin of data plotted in Figure 8.

**Figure 8:**
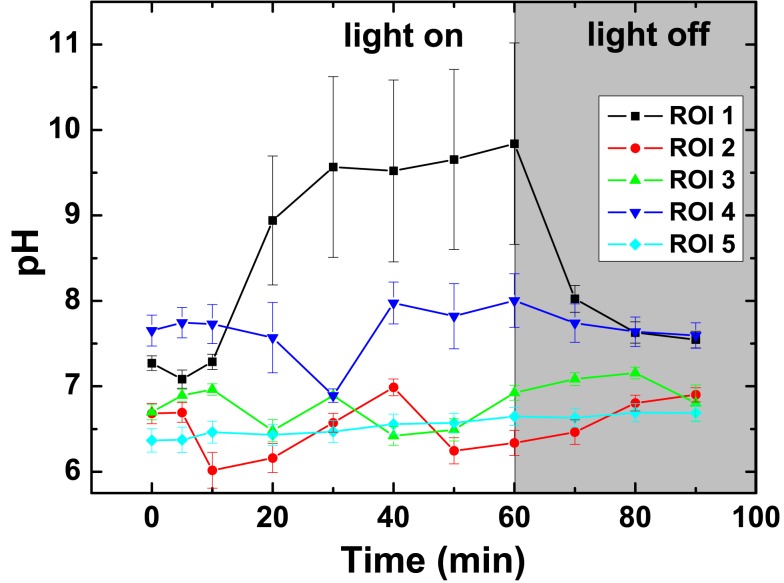
**Dynamics of pH in selected regions of interest (ROI; see Figure 7) after on set of illumination with a photon irradiance of 250 μmol photons m^−2^ s^−1^ and after darkening**. Symbols represent the average ± standard deviation in each ROI.

The pH dynamics in *L. patella* showed pronounced spatio-temporal pH variations and distinct differences to the pH in the surrounding seawater (Figures 7 and 8). The layers containing *Prochloron* in the peribranchial space and common cloacal cavity (e.g., ROI 1 in Figure 7, monochrome image) showed the most pronounced pH dynamics with irradiance, shifting from about pH 7 to 7.5 in darkness to almost pH 10 under an irradiance of 250 μmol photons m^−2^ s^−1^. This shift occurred within ∼20 min in response to a light-dark shift.

Layers in the upper tunic (e.g., ROI2 in Figure 7, monochrome image) and the basal tunic (e.g., ROI3 and ROI5 in Figure 7, monochrome image) showed slightly acidic conditions around pH 6.5–7 both in darkness and during illumination. A patch of cyanobacterial biofilm colonizing the underside of *L. patella* (ROI4 in Figure 7, monochrome image) showed a slightly higher pH reaching pH 7.5–7.8 (with the exception of one time point showing an intermittent dip to pH 7.0), and exhibited no clear light-dark dynamics under the experimental irradiance levels.

The pH images showed some conspicuous hot spots of strong pH dynamics in part reflecting different amounts of *Prochloron* in the cloacal cavities (Figure 7). However, the surrounding host tissue also exhibited an interesting pattern of fluctuating pH, e.g., initially becoming more acidic upon onset of illumination and then exhibiting fluctuating yet acidic pH conditions (Figure 7, light on 0–60 min). Such fluctuations and hot spots were also evident after darkening, where the host tissue in some regions of the upper test apparently became less acidic (Figure 7, light off 10–30 min).

## Discussion

Application of microsensors and bioimaging revealed new and, up to now, the most detailed insights to the microenvironment of *Prochloron* and its host *L. patella* and confirmed preliminary data obtained in the didemnid ascidian *Diplosoma virens* (Kühl and Larkum, 2002) and the first few light and O_2_ measurements in *L. patella* obtained during a survey of its microbiome (Behrendt et al., 2012a). In addition to more detailed microsensor measurements of O_2_ and scalar irradiance, this study presents the first use of O_2_ and pH imaging in photosymbiotic ascidians and combines this new methodology with variable chlorophyll fluorescence imaging of light absorption and PSII activity at similar high spatio-temporal resolution. This allowed us to map both the dynamic chemical landscape and the distribution of photosynthetic activity onto the structural heterogeneity of *L. patella* and its photosymbionts. In the following, we discuss the implications of these new microenvironmental analyses in light of what is currently known about the physiology and diversity of photosymbionts in the *Prochloron*-didemnid ascidian association and its associated microbiome.

### Optical properties and light propagation

Microscale light measurement showed that both visible and NIR were scattered strongly in the opaque tunic of *L. patella* and such local photon trapping led to local maxima in scalar irradiance significantly above the incident downwelling irradiance, i.e., up to 170 and 230% for VIS and NIR, respectively. Similar photon trapping has been observed in different optically dense biological systems with pronounced scattering like sediments, biofilms, plant, and animal tissue (e.g., Vogelmann and Björn, 1986; Vogelmann, 1993; Kühl et al., 1994, 1995; Magnusson et al., 2007). The magnitude of the scalar irradiance maximum is modulated by the optical properties of the system, especially the refractive index, the absorption and scattering coefficients as well as the characteristic scattering phase function of the matrix (see details in Kühl and Jørgensen, 1994).

The optical properties of the tunic matrix are largely unknown but with the exception of the genus *Diplosoma*, photosymbiotic didemnids are known to contain scattering spheroid spicules in their tunic. It has been speculated that these calcareous spicules could have an important role for light propagation and photoadaptation in the tunic of photosymbiotic ascidians (Hirose et al., 2006). A role for spicules in light guiding has been demonstrated in sponges harboring photosymbionts, where radially arranged bundles of spicules channel light into deeper layers of the sponge (Brümmer et al., 2008). We did not see any evidence for light guiding in *L. patella* when imaging cross-sections of a colony, while irradiating it from above. However, it is known from other tissues, such as leaves (Gorton et al., 2010) and corals (Wangpraseurt et al., 2012), that direct and diffuse light can have very different results on light scattering in internal tissues.

Moreover, spicules in photosymbiotic ascidians do not form bundles and can be regarded amorphous scattering particles distributed in the tunic – often at higher density in the upper layers. Hirose et al. (2006) studied the *Prochloron*-containing ascidian *Didemnum molle*, which showed a decreasing density of spicules in the tunic with increasing water depth, and they proposed that high spicule density in shallow water primarily induced photoprotection by increasing the reflectivity of the ascidians. We are not aware of similar studies of the spicule density in *L. patella*, but our light measurements showed pronounced scattering in the upper tunic leading to photon trapping and local amplification of scalar irradiance, especially in the NIR region, where absorption in the tunic is minimal and such scattering effects therefore are most pronounced (Figure 3).

Increased photon pathlength due to spicule-induced scattering in the tunic is but one of several mechanisms potentially affecting light levels and photon trapping. The refractive index of the tunic matrix relative to that of the surrounding seawater can also play an important role as refractive index mismatches can enhance redirection of upwelling scattered light back into the tunic and further enhance photon pathlengths of scattered light (Kühl and Jørgensen, 1994).

A somewhat similar mechanism, assuming absence of scattering in the tissue layer, has been proposed in corals, where multiple scattering and enhanced diffuse reflectance from the coral skeleton underneath leads to a more efficient absorption of incident light by photopigments (Enriquez et al., 2005). In the case of completely diffuse backscatter, the average path length of upwelling photons is twice that of photons in a collimated light beam traversing a thin layer of tissue (Kühl and Jørgensen, 1994). This implicates that the probability for a photon to become absorbed is significantly enhanced, as long as the density of absorbers is not so high that self-shadowing and package effects arise. As a result of this phenomenon, corals inhabiting high light environments can maximize their absorption capacity with low pigment investment while reducing self-shading (Enriquez et al., 2005). Similar effects may be at play in the spicule-containing tunic of photosymbiotic ascidians.

All the above mentioned light trapping and scattering mechanisms lead to enhanced spectral filtering as an increased photon path length due to scattering increases the probability for encountering absorbing pigments in the tunic. Accordingly, photons at wavelengths within major absorption bands of host and photosymbiont pigments will be absorbed more efficiently, while photons outside such absorption bands will be unaffected leading to larger relative differences in the light spectrum. This can have important implications for the *Prochloron*-ascidian association, where UV-screening mycosporine like aminoacids (MAAs) have been found in the upper tunic (e.g., Hirose et al., 2004). Genomic analyses show that such compounds can be synthesized by *Prochloron* (Donia et al., 2011b) but the translocation to the ascidian host tissue remains to be studied, and *Prochloron* may not be the only source. We hypothesize that the presence of spicules and the scattering properties of the upper tunic enable efficient UV protection in analogy to similar effects in corals (Enriquez et al., 2005), while at the same time ensuring that sufficient visible wavelengths can propagate into deeper layers with *Prochloron*. Interestingly, we also found a higher density of spicules in the tunic immediately below the *Prochloron* layer, which may further affect the light field and spectral absorption in *L. patella*, but more detailed optical studies are needed to test such hypotheses.

### *In vivo* conditions and photosynthesis of *Prochloron*

Our microenvironmental measurements show that *Prochloron*, in the peribranchial space and cloacal cavity, inhabits a very dynamic ecological niche in *L. patella*, wherein chemical conditions respond rapidly to changes in irradiance over a timescale of 15–30 min. In the case of O_2_, levels changed quickly from anoxic in the dark up to >250% O_2_ air saturation at PAR irradiances >300 μmol photon m^−2^ s^−1^ (Figure 4B). The O_2_ imaging approach supported these results and in addition allowed a much more detailed assessment of the contributions of other oxygenic photosymbionts in *L. patella*.

The pH imaging system revealed that the pH in the peribranchial/common cloacal cavity shifted from ∼pH 7.0 to pH 7.5 in the dark to just below pH 10.0 under a PAR irradiance of ∼250 μmol photon m^−2^ s^−1^. The pH in this zone is thus lower than in seawater (∼pH 8.2) in the dark and much higher under moderately high irradiance. As the peribranchial/common cloacal cavities are connected to the external seawater by inhalant siphons, one per branchial cavity, it is clear that the internal environment is strongly influenced by the ascidian tissues and *Prochloron* both in the dark and in the light. While photosynthesis can proceed at >pH 9.5 (e.g., Giordano et al., 2005) it is entirely dependent on ${\text{HCO}}_{3}^{-}$ at that pH, since CO_2_ is absent under such alkaline conditions. This suggests that inorganic carbon transport in *Prochloron* is mainly ${\text{HCO}}_{3}^{-}$-dependent. This conclusion is consistent with the evidence from a recent genomics study (Donia et al., 2011b) showing that *Prochloron* only has low-affinity carbon transport pumps. *Prochloron* also lacks many transporters found in other cyanobacteria, including those involved in high-affinity CO_2_ uptake (NdhD3-F3) and the low-CO_2_ inducible bicarbonate transporters *sbtA* and *CmpA-D* (Badger et al., 2002). Interestingly, the upper and lower tunic layers that showed some evidence of O_2_-evolving cyanobacteria were acidic in the light, possibly reflecting a much greater influence of surrounding ascidian cells. The slightly acidic tissue adjacent to the peribranchial/common cloacal cavities and the local hotspots also reflected changes in the animal host tissues, probably in response to the massive change in pH of the peribranchial/common cloacal cavities.

The data presented here for the first time show *in vivo* photosynthesis of *Prochloron* in the peribranchial/cloacal cavity to be both high and efficient (Figures 2–4). Early work on freshly extracted *Prochloron* (Critchley and Andrews, 1984) indicated that photosynthetic O_2_ evolution was very efficient [up to 180 μmol O_2_ (mg Chl)^−1^ h^−1^] under irradiances of 300–400 μmol photons m^−2^ s^−1^, supported by *in vivo* measurements of variable chlorophyll fluorescence (Schreiber et al., 1997). The direct measurements here are similar (Figure 2) and the light measurements suggest that in shallow reef waters *Prochloron* can experience irradiance levels of >300–400 μmol photons m^−2^ s^−1^ *in hospite*. It is noteworthy that in our variable chlorophyll fluorescence imaging, we used blue actinic light exposing a cut surface of *L. patella* evenly, so that the filtering effect of the upper tunic was avoided. The apparent lack of photoinhibition indicates that even at higher irradiance photosynthesis would not be impaired. Our data are in line with earlier observations on high irradiance tolerance of photosynthesis in *Prochloron* when associated with its didemnid host (e.g., Alberte et al., 1987; Lewin and Cheng, 1989). The presence of UVA/B absorbing MAAs in the outer tunic, shown in previous work (e.g., Hirose et al., 2004) and by the *in vivo* spectra here (Figure 3B), could be an important factor allowing high photosynthetic rates of *Prochloron* *in situ* even at high ambient solar irradiance. Furthermore, Lesser and Stochaij (1990) demonstrated the presence of antioxidant enzymes such as superoxide dismutase, ascorbate peroxidase, and catalase in the *Prochloron*-*L. patella* symbiosis, and showed that the activity of these enzymes were directly proportional with irradiance. *Prochloron* thus seems well adapted to quench ROS formed in the photosynthetic apparatus under high irradiance. This is further supported by the presence of multiple *hli* genes in the genome of *Prochloron* (Donia et al., 2011b). These genes encode so-called high light induced proteins that are thought to play a role in quenching of excess excitation energy (He et al., 2001).

**Figure 9:**
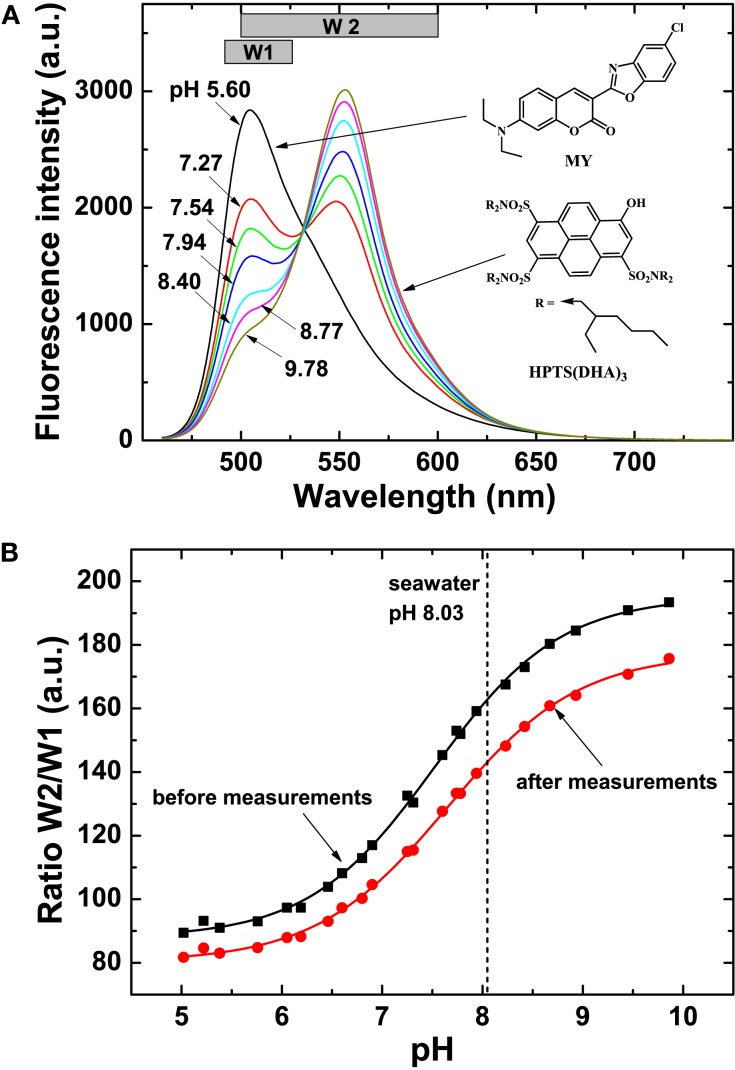
**Spectral characteristics and calibration of sensor foils used for pH imaging**. **(A)** Spectral properties of the pH sensor material showing emission spectra of the pH planar optode (λ_exc_ = 450 nm, 0.6 M NaCl, 25°C) at varying pH (indicated on curves), chemical structures of the dyes and the windows (W1 and W2) used for the interrogation with a multispectral imaging system. The spectra were obtained on a Hitachi F-7000 fluorescence spectrometer. **(B)** Calibration curves of the pH sensor foil, i.e., a plot of the ratio of W2/W1 vs. defined pH levels. Note that the optimal dynamic range of the optode is at pH 6–9. The sigmoidal calibration curve flattens at pH >9 and <6, and pH values obtained in these ranges are thus less accurate.

As pointed out in the above section, under these high photosynthetic conditions, the O_2_ concentrations and pH in the peribranchial/cloacal cavity rise to very high levels (Figures 5–9). Clearly neither of these factors inhibits the photosynthetic capacity of *Prochloron* and as pointed out above, the low-affinity inorganic carbon transporters in the plasma membrane of *Prochloron* must be able to transport adequate quantities of ${\text{HCO}}_{3}^{-}$ into the cells. Whether any role is played by matrix compounds present in the *Prochloron* layer (Figure 1), and whether the host exerts any control on the uptake of inorganic carbon across the plasma membrane (Critchley and Andrews, 1984; Griffiths, 2006) are important questions for future research.

### Other oxyphototrophs in didemnid ascidians

Besides *Prochloron*, other phototrophs occur in the upper unic of *L. patella* and as judged from the *in vivo* spectral light measurements (Figure 3) appear to be phycobilin-containing cyanobacteria. This is in line with the findings in a recent survey of the microbial diversity in *L. patella*, where *16S RNA* gene sequences and hyperspectral signatures of cyanobacteria were abundant in the upper tunic (Behrendt et al., 2012a). Another cyanobacterial zone also lies in the tunic beneath the *Prochloron* layer, the photosynthetic activity of which became evident in the imaging of PSII activity, O_2_ and pH. The slower dynamics of O_2_ in this layer is probably the result of light screening by cyanobacteria in the upper test and the *Prochloron* layer. Both zones of cyanobacteria have been observed before and the tunic is known to harbor the cyanobacterium *Synechocystis trididemni* and other cyanobacteria with special pigmentation (Lafargue and Duclaux, 1979; Kott, 1984; Cox et al., 1985; Hirose et al., 2009b; López-Legentil et al., 2011). López-Legentil et al. (2011) found Chl *d*-containing *Acaryochloris cells* in the unic of other didemnid ascidians on mangrove roots in the Bahamas but we have not found any evidence for Chl *d* in the unic of *L*. *patella* (Kühl et al., 2005; Behrendt et al., 2012a).

The other oxyphototrophs in *L. patella* are exposed to very different irradiance regimes in comparison to *Prochloron*. Those colonizing the surface and upper tunic would be exposed, at times, to high and potentially photoinhibitory irradiance levels reaching >400 μmol photons m^−2^ s^−1^. In contrast, *S. trididemni* and other cyanobacteria below *Prochloron* layer would be much better shielded and prone to light limitation at high irradiance as a result of the strongly absorbing *Prochloron* layer above (Figure 3). Due to strong light attenuation and spectral filtration by *Prochloron*, the cyanobacteria thriving below it have adapted their pigmentation and *S. trididemni* and other cyanobacteria present contain phycourobilin in addition to other more conventional phycobiliproteins (Cox et al., 1985; Hirose et al., 2009b), which would allow them to harvest green light very efficiently; it is probably phycourobilin that contributes to the high absorption at around 500 nm in the deeper tunic layers (Figure 3B; cf Hirose et al., 2009b). In the present study, we did not specifically target the identification and abundance of these cyanobacteria in the tunic of *L. patella*. Behrendt et al. (2012a) also did not extend their microbial diversity survey to the inhabitants of the lower tunic in *L. patella*; however, our data point to a rather dense and active community, which awaits further investigation.

Our imaging studies also revealed the presence of oxyphototrophs in the biofilm layer colonizing the underside of *L. patella*. This biofilm is highly diverse, with cyanobacteria as the predominant oxyphototrophs, especially *A. marina* that thrives in this ecological niche deprived of visible wavelengths but with abundant NIR supporting its Chl *d*-based oxygenic photosynthesis (Kühl et al., 2005; Behrendt et al., 2012a; Larkum et al., 2012). The present results thus confirm our previous studies (Kühl et al., 2005, 2007a) showing active photosynthesis in this biofilm albeit with a more moderate dynamics in both O_2_ and pH as compared to *Prochloron*. Actually, both the O_2_ and pH imaging data represent the very first *in vivo* measurements of these parameters in the natural habitat of *A. marina*, but a detailed discussion of the implications of these observations for our knowledge on the *in situ* biology of Chl *d*-containing cyanobacteria is beyond the scope of this study.

### The microbiome of *L. patella*

Sequencing of the *16S rRNA* gene and bulk metagenomic analysis have provided detailed information on the composition of a tremendous variety of microbial assemblages, ranging from the human body to marine environments. Similar studies performed on ascidian-associated microbial communities revealed a high bacterial diversity (Martínez-García et al., 2007; Donia et al., 2011a; Behrendt et al., 2012a), possibly due to distinct microenvironments as demonstrated in this study, and domination by selected phyla such as proteobacteria and cyanobacteria (Tait et al., 2007; Menezes et al., 2010; López-Legentil et al., 2011; Behrendt et al., 2012a). Combined molecular and microenvironmental measurements on *L. patella* demonstrated the coexistence of three very different microbial communities, separated by only a few millimeters of animal tissue (Behrendt et al., 2011): (i) a biofilm on the upper surface exposed to high irradiance, (ii) a cloacal cavity dominated by *Prochloron* spp. characterized by strong depletion of VIS and a dynamic chemical microenvironment, and (iii) a biofilm community covering the underside of the animal, where light is depleted of visible wavelengths and enriched in NIR. Further molecular analysis of the *L. patella* microbiome revealed bacteria with a rich genetic diversity, capable of producing significant amounts of secondary bioactive metabolites (Donia et al., 2011a) and earlier studies highlighted *Prochloron* as one of the key producers of such compounds (Schmidt et al., 2005). Further metagenomic investigations of *Prochloron* cells revealed an arsenal of functional gene cassettes coding for secondary metabolites across large geographical distances, with surprisingly little genomic diversion within the different *Prochloron* cells (Donia et al., 2011a). Even though *Prochloron* seems to functionally depend on its host, its genome contains a set of core genes similar to other cyanobacteria (Donia et al., 2011b), which, in all likelihood allows life outside of the ascidian-*Prochloron* association. Despite this, larger amounts of free-living *Prochloron* cells have only been reported once (Cox, 1986), while facultative relationships have been reported between non-ascidian invertebrates and *Prochloron* cells (Cheng and Lewin, 1984; Parry, 1986).

The abundance of *Prochloron* cells appears to underlie seasonal changes in temperature (McCourt et al., 1984) and more generally the distribution of cyanobacteria is found to be governed by the composition and availability of light (Stomp et al., 2007a,b). NIR-utilizing bacteria associated with ascidians are a prime example of such a highly specialized niche-partitioning: the Chl *d*-containing cyanobacterium *A. marina* was found to survive below didemnid ascidians in a light environment relatively enriched in NIR and depleted in VIS due to the overlying layer of Chl *a*/*b*-containing *Prochloron* cells (Kühl et al., 2005, 2007a; Behrendt et al., 2012a). Such microenvironmental patterns can even be extrapolated to larger biological frameworks: the sampling of *L. patella* associated biofilms along a depth gradient thus revealed a negative correlation between the abundance of NIR-utilizing phototrophs (*A. marina, Rhodospirillaceae*, *Rhodobacteraceae*, *Chloracidobacteria*) and water depth indicating that their abundance was strongly influenced by the availability of NIR, which is readily attenuated by seawater (Behrendt et al., 2012a). This highlights the need to perform ecologically relevant molecular surveys of microbial diversity on an appropriate (micro-) scale with accompanying metadata. In this context, microenvironmental data can inform both observations obtained by genomics and even the actual planning of such surveys. We have demonstrated that *Prochloron* lives in a niche experiencing strong fluctuations of the chemical microenvironment, generally experiencing hypoxia or anoxia inside their ascidian host under low light or dark conditions. Such microenvironmental settings thus point to a potential presence of a number of anaerobic processes and microbes.

In densely populated cyanobacterial communities experiencing periods of hypoxia/anoxia, N_2_ fixation is often found (Stal and Zehr, 2008; Steunou et al., 2008). Diazotrophy has been reported several times in photosymbiotic ascidians, but whether *Prochloron* can fix N_2_ remains unclear (Paerl, 1984; Koike et al., 1993; Kline and Lewin, 1999). Interestingly, genes associated with N_2_ fixation were absent in the recently published *Prochloron* genome, apparently forcing it to rely on recycled nitrogen from its host via a number of nitrogen transformation pathways present in the genome (Donia et al., 2011b). This fits with the observation that ammonium is the major nitrogenous waste product of the ascidian host (Goodbody, 1974), which is also effectively taken up by *Prochloron* (Parry, 1985), while the presence of genes for urease and urea uptake point toward a potential role of urea in the nitrogen cycling between host and symbiont (Donia et al., 2011b). Diazotrophy may, however, occur in *Prochlorons* microenvironment as other N_2_-fixing bacteria such as *Azospirillum brasilense* were found to reside in the cloacal cavity of *L. patella* (Behrendt et al., 2012a) and this may explain some of the earlier observations of N_2_ fixation (Paerl, 1984). Nevertheless, *Prochloron* is clearly the predominant inhabitant of the peribranchial space and inner cloacal cavities of *L. patella*, and we speculate that such diazotrophs may benefit from the strong O_2_ dynamics imposed by *Prochloron*.

It remains to be investigated what type of dark metabolism *Prochloron* is thriving on during periods of low or no light and anoxia. Our data show that oxic dark respiration of *Prochloron* is limited due to strong O_2_ depletion in its microenvironment. Under such conditions, cyanobacteria can rely on fermentation for their energy metabolism (Stal and Moezelaar, 1997). To our knowledge, the presence and activity of fermentative pathways in *Prochloron* have not been investigated. A first look into the *Prochloron* genome published by Donia et al. (2011b) actually revealed the potential presence of both mixed acid as well as lactate/pyruvate fermentation pathways (data not shown), which are known to occur in cyanobacteria (Stal and Moezelaar, 1997). Our finding of low pH in darkness support the speculation that fermentation may be an important part of *Prochloron* dark metabolism. Confirmation of this and further insight to the dark metabolism of *Prochloron* awaits more dedicated physiological studies of its carbon metabolism, preferentially in combination with further scrutiny of the *Prochloron* genome and subsequent transcriptome analyses. The same holds true for other aspects of *Prochloron* metabolism and biosynthesis, such as the synthesis of UV protective MAAs, abundant lipids, sterols, and bioactive secondary metabolites in *L. patella* (Donia et al., 2011a,b). While the presence of these pathways in *Prochloron* has been shown, we still know nothing about the diel dynamics of gene expression and enzyme synthesis in photosymbiotic ascidians and how particular microenvironmental controls play a regulatory role. A combination of microenvironmental analysis with genomics approaches (see, e.g., Steunou et al., 2008; Jensen et al., 2011) to further resolve the biology of *Prochloron* thus seems a very promising way to proceed.

*Prochloron* remains uncultivated despite numerous attempts since its discovery in 1975 (Hirose et al., 2009a). An hitherto unconfirmed report of successful cultivation argued that *Prochloron* lacked the ability to synthesize tryptophan (Patterson and Withers, 1982), while the recent *Prochloron* genome shows the presence of a full set of tryptophan anabolic genes (Donia et al., 2011b). Interestingly, Patterson and Withers (1982) also found cell division of *Prochloron* under acidic conditions with maximal growth at pH 5.5 under low irradiance of ∼110 μmol photons m^−2^ s^−1^ over a 18:6 h light-dark cycle. We found acidification of the *Prochloron* microhabitat in response to darkness, albeit only a few areas reached pH <6 (see, e.g., Figures 7C,D). In contrast, *Prochloron* photosynthesis is limited to a higher pH range with a maximum at pH 8–8.5 (Dionisio-Sese et al., 2001). Not much is known about the cell division cycle of *Prochloron*, besides one study reporting a diurnal rhythm with maxima of dividing cells in early morning and afternoon (Lewin et al., 1984), and we do not know how such cell division pattern is modulated by microenvironmental changes.

With the new knowledge on pH and O_2_ dynamics presented here, it is possible to further optimize enrichment and cultivation scenarios better mimicking the natural habitat of *Prochloron* and to test new working hypotheses such as the importance of chemical and light gradients for *Prochloron* growth, and the importance of keeping *Prochloron* in a biofilm/aggregated growth mode for longer survival outside its host. A promising approach for enrichment and growth of *Prochloron* cells in gradients under controlled biofilm-like conditions could be based on immobilization of extracted *Prochloron* cells in alginate beads; an approach recently shown to work with *A. marina* (Behrendt et al., 2012b).

## Materials and Methods

### Field site and sample collections

Didemnid ascidians were sampled at low tide on the outer reef flat and crest off Heron Island (S23°26′0055, E151°55′0850), Great Barrier Reef in the Austral summer (January–February) during several field trips in the period 2001–2012. Specimen of *L. patella* were sample as larger intact specimens (5–20 cm^2^; 5–20 mm thick) covering coral patches on the outer reef flat and down to ∼4 m depth on the reef crest (Figure 1). A more detailed description of sampling and field sites is given in Behrendt et al. (2012a).

Collection of didemnids was either done by hand or snorkeling. The samples were immediately transported in a bucket with sea water back to Heron Island Research Station, where they were transferred to outdoor aquaria continuously flushed with fresh aerated seawater (26–28°C) pumped in from the reef. Frequent exchange of water by flushing and continuous strong mixing of water in the aquarium is necessary to avoid degradation of the ascidians. To avoid high solar irradiance, the aquaria were covered by shading cloth dampening solar irradiance to ∼200–300 μmol photons m^−2^ s^−1^. Under such conditions we could keep the sampled ascidians healthy and with actively photosynthesizing *Prochloron* for up to a week.

Measurements were done on small intact colonies of *L. patella* (∼2–4 cm^2^, 5–10 mm thick), as well as on specimens with an exposed fresh vertical cut through the test or, alternatively, on 1–2 mm thick vertical sections of *L. patella*. Transfer, slicing and cutting of specimen were done in a large beaker of seawater to buffer pH changes due to release of acid vacuoles. Relatively flat and homogeneous pieces of *L. pa*tella with a surface area of a few cm^2^ were cut with a scalpel and immediately rinsed and submerged in filtered seawater. Cross-sections were cut from homogenous pieces with a razor blade for subsequent imaging. Generally, specimens showed a high maximal PSII quantum yield of >0.7 for several hours after such handling indicative of fast recovery and minimal stress on *Prochloron*.

### Electron microscopy

#### TEM

Specimens were fixed with 2% v/v glutaraldehyde in 0.05 M sodium phosphate buffer (pH 7.2). Following isolation of suitable specimen blocks, the samples were rinsed three times in 0.15 M sodium cacodylate buffer (pH 7.2) and subsequently postfixed in 1% w/v OsO_4_ in 0.12 M sodium cacodylate buffer (pH 7.2) for 2 h. The specimens were dehydrated in graded series of ethanol, transferred to propylene oxide, and embedded in Epon according to standard procedures. Sections, ∼80 nm thick, were cut with a Reichert-Jung Ultracut E microtome and collected on copper grids with Formvar supporting membranes. Ultra thin sections were collected on copper grids with Formvar supporting membranes and stained with uranyl acetate and lead citrate, and subsequently examined with a Philips CM 100 TEM (Philips, Eindhoven, Netherlands), operated at an accelerating voltage of 80 kV and equipped with an OSIS Veleta digital slow scan 2k × 2k CCD camera. Digital images were recorded with the ITEM software package.

#### FIB (focused ionbeam) SEM

Specimens were fixed with 2% glutaraldehyde in 0.05 M phosphate buffer (pH 7.2) and postfixed in 1% w/v OsO_4_ with 1.5% potassium ferrocyanide. Following a rinse in dH_2_O the specimens were stained en bloc in 1% Uranyl acetate over night, dehydrated in ethanol, and embedded in Epon according to standard protocols. The Epon blocs were mounted on aluminum stubs with colloidal carbon as an adhesive, sputter-coated with gold (Polaron SEM Coating Unit E5000), and imaged with a Quanta 3D FEG (FEI) operated at 5 kV using a vCD backscattered electron detector. Additionally, mounted cross-sections of the same samples were imaged on a Tecnai G2 20 Twin transmission electron microscope.

### Imaging of variable chlorophyll fluorescence

Cross-sections through *L. patella* were placed in a small Petri-dish and covered by a thin (∼1–2 mm) layer of seawater and investigated with a variable chlorophyll fluorescence imaging system (I-PAM, Walz GmbH, Germany) consisting of a CCD camera, a LED ring light (with blue, red, and NIR LEDs), and a controlling unit connected to a PC running the dedicated imaging system software (Imaging-WIN 2.3, Walz GmbH, Germany). The blue LEDs (470 nm) provided both weak pulses of measuring light as well as defined levels of actinic light measured as downwelling photon irradiance, *E*_d_, in the focus plane of the system with a calibrated irradiance meter (LI-250 and LI-192, LiCor, USA). A detailed description of variable chlorophyll fluorescence imaging systems is given elsewhere (Grunwald and Kühl, 2004; Ralph et al., 2005; Kühl and Polerecky, 2008; Trampe et al., 2011). The system enabled quantification of a proxy for PAR absorptivity, A, by measuring the reflected red light (*R*, 650 nm) and NIR (*R*_NIR_, 780 nm) from the specimen/sample and calculating the ratio *A* = 1 − (*R*/*R*_NIR_).

Based on the non-actinic imaging of the chlorophyll fluorescent yield before and during a strong saturation pulse, using weak modulated blue measuring light, several parameters characterizing the distribution and photosynthetic performance of oxyphototrophs in the ascidian specimens could be quantified and visualized. This included:

(1) The maximal quantum yield of PSII electron transport, Φ_max_ = (*F*_m_-*F*_0_)/*F*_m_, where F_0_ is the minimal fluorescence yield and *F*_m_ is the maximal fluorescent yield of the dark-adapted sample measured prior to and during a strong saturation pulse, respectively;
(2) The effective quantum yield of PSII electron transport $\Phi_{\text{PSII}}=\left(F_{\text{m}}^{\prime }-F\right)∕F_{\text{m}}^{\prime }$, where *F* is the fluorescent yield under a known level of blue actinic light and $F_{\text{m}}^{\prime }$ is the maximal fluorescent yield during a subsequent saturation pulse, respectively.

Based on these measurements, we calculated images of a range of derived photophysiological parameters characterizing photochemical and non-photochemical quenching of absorbed light energy in *Prochloron* and other oxyphototrophs present in the ascidians: a proxy for the relative photosynthetic activity was calculated from the relative PSII electron transport rate rETR = Φ_PSII_ *E*_d_ by taking the different absorptivity over the imaged specimen into account, as PS = *A* Φ_PSII_ *E*_d_.

The imaging system enabled acquisition of the mentioned parameters under a range of different actinic light levels applied to the samples over pre-defined time periods ranging from 10–20 s to 10 min. In this way, we could obtain information on both rapid and slow responses of photosynthetic performance. So-called rapid light curves (RLC) were obtained my measuring Φ_PSII_ at increasing irradiance using 10–20 s exposure to each irradiance level. Such light curves provide a snap-shot of the current photosynthetic capacity for handling light but should not be regarded similar to traditional photosynthesis vs. irradiance curves that are measured at steady state after longer incubation times (Schreiber et al., 1997). Such steady state light curves (SLC) of Φ_PSII_ vs. irradiance were obtained using 5–10 min long incubation times. Besides images, information averaged over particular regions of interest (ROI) was extracted with the system software (Image WIN 2.3, Walz GmbH, Germany).

### Microscale light measurements

Light propagation in the ascidian tissues was measured with fiber-optic scalar irradiance microprobes (Kühl, 2005) coupled to a fiber-optic spectrometer (QE65000, Ocean Optics, Dunedin, USA) for measurements of spectral scalar irradiance. Intact ascidian samples were fixed onto a black neoprene holder by thin insect preparation needles and placed in a flow chamber. The scalar irradiance microprobe was mounted in a manually operated micromanipulator (MM33, Märzhäuser, Wetzlar, Germany) and inserted into the ascidian tissue at an angle of 45° relative to the vertically incident light from a fiber-optic halogen lamp equipped with a collimating lens (KL2500, Schott, Germany). The solid test material of *L. patella* did not allow direct insertion of the spherical microprobe tip, and it was necessary to make a minute incision with the tip of a thin (29G) hypodermic needle prior to measurements.

All scalar irradiance measurements were normalized to the incident downwelling irradiance as measured with the scalar irradiance microprobe positioned in a black light well at a similar position in the light field as over the unic surface. Spectral measurement in different depths were also integrated over visible wavelengths (400–700 nm) driving oxygenic photosynthesis in *Prochloron* as well as over a region in the NIR (700–740 nm) that can be used by oxyphototrophs containing Chl *d* or *f*. This allowed calculation of depth profiles of VIS and NIR scalar irradiance in % of the incident downwelling irradiance in the respective spectral ranges.

### Microscale O_2_ and pH measurements

Microscale O_2_ measurements were done with amperometric Clark-type O_2_ microelectrodes (Revsbech, 1989) connected to a pA-meter (PA2000 or Microsensor Multimeter, Unisense, Denmark). The O_2_ microsensors exhibited a fast response time (*t*_90_ < 0.5 s), low stirring sensitivity (<1–2%) and had tip diameters of 25–50 μm. In *L. patella*, it was necessary to make an incision with a thin (29G) hypodermic needle prior to measurements. The O_2_ microsensors were linearly calibrated from sensor readings in O_2_-free solution (seawater amended with sodium dithionite) and in aerated seawater at experimental temperature and salinity (100% atmospheric saturation).

Microscale pH measurements were attempted with pH glass needle electrodes (pH-N, Unisense AS, Aarhus, Denmark; Kühl and Revsbech, 2001) connected to a high impedance mV meter (Keithley, USA) and calibrated at experimental temperature in standard pH buffers at pH 4, 7, and 10. The sensors exhibited a near-ideal Nernstian response of 55–59 mV per pH unit change. However, the pH sensors were too fragile for profiling pH in the sturdy tunic of *L. patella* and we observed apparent local release of acid vacuoles, when inserting the relatively large needle sensors, that changed the pH microenvironment dramatically down to pH 2–3 within seconds before slowly rising again to ambient pH over some minutes.

For microprofiling, the microsensors were mounted in a PC-controlled motorized micromanipulator system (Unisense, Denmark) that enabled automated profiling (in vertical steps of 50–100 μm) and data acquisition via dedicated software (Profix, Pyroscience, Germany).

### Imaging of O_2_ and pH distribution and dynamics

The spatial distribution and dynamics of O_2_ concentration and pH in *L. patella* was imaged on a vertical cut specimen, which was mounted tightly up against the transparent flow chamber wall with help of two hypodermic needles fixed in the soft chamber bottom. The planar optodes (see below) were positioned between the specimen and the inner wall of the flow chamber. The temperature in the flow chamber was kept constant at 26°C. Actinic light was provided vertically from above using a fiber-optic halogen lamp (LG-PS2, Olympus, Japan). To avoid interference, the lamp was briefly switched off during the image acquisition. The irradiance of the actinic light at the level of the ascidian test surface was determined in water with a scalar irradiance mini sensor (Model US-SQS/L, Walz GmbH, Effeltrich, Germany) coupled to a calibrated irradiance meter (ULM-500, Walz GmbH, Effeltrich, Germany). Photographic images of the vertically cut ascidian surface mounted against the transparent flow chamber were taken with a digital camera (Canon 5D, Canon, Japan).

#### Sensor materials

The fundamentals of sensor fabrication and solute imaging with planar optodes as well as details of sensor synthesis and physico-chemical properties are described in detail elsewhere (e.g., Borisov et al., 2008, 2009a,b; Kühl and Polerecky, 2008; Kühl et al., 2007b, 2008; Mayr et al., 2009; Larsen et al., 2011; Staal et al., 2011; Fabricius-Dyg et al., 2012). Planar optodes for O_2_ imaging were prepared by first dissolving 3 mg of platinum(II) tetra(4-fluoro)phenyltetrabenzoporphyrin (=PtTPTBPF; Borisov et al., 2008, 2009a) and 200 mg of polystyrene (MW 250,000, Aldrich, USA) in 1.8 g of chloroform. This “cocktail” was knife-coated onto a transparent poly(ethylene terephthalate) support foil (Mylar, Goodfellow, USA). The thickness of the sensing layer after evaporation of the solvent was ∼2.5 μm. The O_2_ sensor chemistry possesses exceptional brightness due to high molar absorption coefficients and luminescence quantum yields of the indicators and enables excitation with red light (λ_max_ 615 nm). The NIR emission at ∼765 nm allows minimization of scattering effects and interfering chlorophyll fluorescence.

Planar optodes for pH imaging were prepared by first dissolving 3 mg of the coumarin dye Macrolex^®^ Fluorescent Yellow (=MY; Simon and Werner GmbH)^1^, 3 mg of lipophilic 1-hydroxypyrene-3,6,8-tris-bis(2-ethylhexyl)sulfonamide [=HPTS(DHA)_3_; Borisov et al., 2009b] and 200 mg of Hydrogel D4 (Cardiotech)^2^ in 1.8 g of ethanol:water (9:1 v/v). The pH sensor “cocktail” was also coated onto a transparent Mylar support foil. The thickness of the pH sensing layer after evaporation of the solvent was ∼7.5 μm.

Since the absorption spectrum of the basic form of the pH indicator perfectly matches the emission spectrum of the MY reference dye, Förster Resonance Energy Transfer (FRET) leads to the emission from HPTS(DHA)_3_ at high pH (Figure 9). The protonated form of HPTS(DHA)_3_ absorbs at about 450 nm, therefore, no FRET is observed at lower pH and the emission from MY is maximal. Thus, the system allows referenced ratiometric imaging of pH by monitoring the emissions from the two dyes – MY at ∼505 nm and HPTS(DHA)_3_ at ∼555 nm.

#### Imaging of O_2_

Planar O_2_ optodes were read out with a lifetime imaging system [see details in Holst et al., 1998) employing a monochrome gated CCD camera (Sensicam-Sensimod, PCO, Kehlheim, Germany)] equipped with a Schneider-Kreuznach Xenoplan 1.4/23 CCTV-Lens and a R-720 long-pass filter (Edmunds Optics, USA) mounted in front of the lens. Two red-orange high power LEDs (617 nm, 1 W, Luxeon, Philips Lumileds, USA) were used for excitation, as controlled by a custom-built PC-controlled pulse-delay generator. A rapid lifetime determination method was used to determine the O_2_-dependent luminescence decay time τ by measuring luminescence intensities in two time windows (1–41 and 26–66 μs, respectively) after the excitation pulse:

(1) $$\tau =\frac{t_{1}-t_{2}}{\ln\left(I_{1}/I_{2}\right)},$$

where *t*_1_ and *t*_2_ represent the time corresponding to the start of the first and the second window, respectively (1 and 26 μs in our setup), and *I*_1_ and *I*_2_ are the luminescence intensities acquired in the respective time windows.

A two point calibration (in air saturated and anoxic seawater, respectively) was used to calibrate the τ vs. O_2_ oxygen response. A 2% w/w aqueous solution of sodium dithionite was used for deoxygenation. The following equation was used to obtain the calibration curve and to convert the measured decay times into pO_2_:

(2) $$\frac{\tau }{\tau_{0}}=\frac{f}{1+K_{SV}\left[O_{2}\right]}+1-f,$$

where *f* = 0.91 (determined from the calibration for the PtTPTBPF/PS optodes obtained in the frequency domain measurements), τ_0_ is the decay time in the anoxic solution (53.2 μs), and *K*_SV_ is the Stern–Volmer constant (0.0196 hPa^−1^) as determined from the decay times at air saturation and under anoxic conditions under experimental temperature and salinity.

We used Matlab 7.10 (Mathworks Inc., USA) to calculate pseudocolor decay time images from the original data and, subsequently pseudocolor O_2_ concentration images.

#### Imaging of pH

The pH optodes were read out with a new spectral camera system (Spectrocam, Ocean Thin Films, USA) consisting of a CCD camera combined with a fast-switching filter wheel (eight filter positions) and equipped with a Distagon TX2.8/25 mm ZF lens (Carl Zeiss AG, Germany). A long-pass glass filter (OG 490, Schneider-Kreuznach GmbH, Germany) was mounted in front of the lens to eliminate interference from the reflected excitation light. The pH optode was excited with blue light (λ_max_ 460 nm, as determined with a fiber-optic spectrometer, USB2000+, Ocean Optics, USA) from a torch (Bluestar, NightSea, Bedford, MA, USA). The emission of the pH sensor was monitored in two spectral windows: “window 1” (filter position with a Spectrocam 475/100 nm band width interference filter) and “window 2” (filter position with a Spectrocam 550/100 nm band width interference filter).

The emission of HPTS(DHA)_3_ was imaged with the 550/100 nm filter. Notably, some emission from MY is also detected in this window (Figure 9A). The emission from MY was detected using the 475/100 nm bandpass filter. Thus, the effective window for monitoring the green fluorescence of MY was 490–525 nm. Subsequently, pH was evaluated by the ratio of the two windows: *R* = (window 2/window 1) × 1200.

The pH optode was calibrated by ratiometric imaging in seawater at experimental temperature and salinity, wherein specific pH values were adjusted by addition of 0.1 M HCL or 1 M NaOH. The seawater pH at different calibration points was measured with a calibrated pH glass electrode connected to a pH meter (UB-10, Denver Instruments, USA). Calibration curves were obtained both for a fresh sensor foil and a sensor foil used in experiments to enable compensation for sensor drift due to dye bleaching. Image calculations and pseudocolor pH distribution images were done in *Matlab* 7.10 (Mathworks Inc., USA) by using individual calibration curves generated for each image. The pH of the seawater (8.03) was used for recalibration.

Calibration curves of sensor foils showed a shift in the intensity ratio after the experiment (Figure 9B), caused, e.g., by bleaching of the pH indicator during irradiation with actinic light. Therefore, the intensity ratio in the regions outside of the ascidian sample (seawater, pH 8.03) was used for recalibration. Thereby, an individual calibration curve was generated for each image and the drift was successfully compensated for. Note that the optimal dynamic range of the optodes is at pH 6–9. The sigmoidal calibration curve flattens at pH >9 and pH <6, and pH values obtained in these ranges are therefore prone to less accuracy.

Information on O_2_ concentration and pH (average ± standard deviation) within particular ROI were extracted within *Matlab*.

## Author Contributions

Michael Kühl, Lars Behrendt, Sergey M. Borisov, Ulrich Schreiber, and Anthony W. D. Larkum designed and performed research; Michael Kühl, Erik Trampe, Klaus Qvortrup, Ulrich Schreiber, Sergey M. Borisov, and Ingo Klimant contributed new reagents/analytic tools; Michael Kühl, and Sergey M. Borisov analyzed data; and Michael Kühl, Lars Behrendt, Sergey M. Borisov, and Anthony W. D. Larkum wrote the paper with editorial help from all co-authors.

## Conflict of Interest Statement

The authors declare that the research was conducted in the absence of any commercial or financial relationships that could be construed as a potential conflict of interest.

## Footnotes

^1^http://www.simon-und-werner.de

^2^http://www.cardiotech-inc.com
